# p53 mutant breast cancer patients expressing p53γ have as good a prognosis as wild-type p53 breast cancer patients

**DOI:** 10.1186/bcr2811

**Published:** 2011-01-20

**Authors:** Jean-Christophe Bourdon, Marie P Khoury, Alexandra Diot, Lee Baker, Kenneth Fernandes, Mustapha Aoubala, Philip Quinlan, Colin A Purdie, Lee B Jordan, Anne-Catherine Prats, David P Lane, Alastair M Thompson

**Affiliations:** 1 Centre for Oncology and Molecular Medicine, Inserm-European Associated Laboratory, University of Dundee, Dundee, DD1 9SY, UK; 2 Institut National de la Santé et de la Recherche Médicale (Inserm), U858, F-31432 Toulouse, France; 3 Université de Toulouse, UPS, Institute of Molecular Medicine of Rangueil, IFR150, F-31432 Toulouse, France; 4 Department of Pathology, Ninewells Hospital and Medical School, Dundee, DD1 9SY, UK; 5 p53 Laboratory (p53Lab), 8A Biomedical Grove, #06-06, Immunos, Singapore 138648, Singapore; 6 Department of Surgical Oncology, MD Anderson Cancer Centre, 1400 Holcombe Boulevard, Houston, TX 77030, USA

## Abstract

**Introduction:**

Normal function of the p53 network is lost in most cancers, often through *p53 *mutation. The clinical impact of *p53 *mutations in breast cancer remains uncertain, especially where p53 isoforms may modify the effects of these *p53 *mutations.

**Methods:**

Expression of p53β and p53γ isoforms, the isoforms identified in normal breast tissue, was detected by reverse transcription polymerase chain reaction from a cohort of 127 primary breast tumours. Expression of p53β and p53γ isoforms was analysed in relation to clinical markers and clinical outcomes (5 years) by binary logistic regression, Cox proportional hazards regression and Kaplan-Meier survival analyses.

**Results:**

p53β and p53γ were not randomly expressed in breast cancer. p53β was associated with tumour oestrogen receptor (ER) expression, and p53γ was associated with mutation of the *p53 *gene. The patient group with the mutant *p53 *breast tumour-expressing p53γ isoform had low cancer recurrence and an overall survival as good as that of patients with wild-type p53 breast cancer. Conversely, patients expressing only mutant *p53*, without p53γ isoform expression, had a particularly poor prognosis.

**Conclusions:**

The determination of p53γ expression may allow the identification, independently of the ER status, of two subpopulations of mutant *p53 *breast cancer patients, one expressing p53γ with a prognosis as good as the wild-type *p53* breast cancer patients and a second one not expressing p53γ with a particularly poor prognosis. The p53γ isoform may provide an explanation of the hitherto inconsistent relationship between *p53* mutation, treatment response and outcome in breast cancer.

## Introduction

The p53 pathway is ubiquitously abnormal in human cancers, either through mutation of the *p53 *gene or via modification of p53 function by interaction with oncogenic cellular or viral proteins [1,2]. Somatic *p53 *gene mutations, found in about 25% of breast cancers, are associated with poor prognosis [3,4]. Patients bearing mutant *p53 *breast cancer have resistance to several chemotherapy agents but may be more sensitive to taxanes, at least in the neoadjuvant setting [5-10]. However, the uncertainties around the relationships between *p53 *mutation, therapeutic response and outcome in breast cancer suggest that additional factors may be involved.

The human *p53 *gene expresses at least nine different p53 protein isoforms containing different domains of the p53 protein (p53, p53β, p53γ, Δ133p53α, Δ133p53β, Δ133p53γ, Δ40p53α, Δ40p53β and Δ40p53γ) as a result of multiple splicing, alternative initiation of translation and internal promoter usage [11-13]. The p53 isoforms are differentially expressed in normal human tissues, with normal breast tissue expressing p53, p53β and p53γ [13]. Abnormal expression of p53 isoforms has been identified in several human cancer types [13-19]. We have previously reported that p53 isoforms such as p53β can interact with p53 and modulate p53 tumour suppressor activity [13,19,20]. Taken together, these findings suggest that the p53 isoforms may play a role in human cancers.

In this report, expression of the p53β and p53γ isoforms is examined in relation to clinical and pathological markers, p53 mutation and disease outcome in a cohort of 127 randomly selected primary breast tumours. The patient group expressing the p53γ isoform had abrogation of the poor prognostic effect associated with *p53 *mutation, with a low risk of cancer recurrence and a survival rate as good as that of the patient group bearing wild-type p53 breast cancer. Conversely, patients expressing only mutant *p53*, without p53γ isoform expression, had a particularly poor prognosis. The p53γ isoform may explain the inconsistent relationship between *p53 *mutation and breast cancer in the literature.

## Materials and methods

### Clinical samples

Previously untreated operable primary breast cancer in 127 Caucasian women (age range, 32 to 89 years; median age, 60 years) with sufficient tumour tissue surplus for diagnostic requirements and complete clinical and pathological data was analysed. Tumour tissues were macrodissected by a specialist breast pathologist and snap-frozen in liquid nitrogen prior to storage at -80°C. Samples were examined following Local Research Ethics Committee approval under delegated authority from the Tayside Tissue Bank. The Tayside Tissue Bank has received ethical approval for its activities (REC Reference 07/S1402/90).

### Reverse transcription-polymerase chain reaction analysis

Approximately 10 mg of tumour tissue (>40% of tumour cells) was homogenised in 750 μL of QIAzol lysis reagent (Qiagen Ltd, Crawley, West Sussex, UK), and total RNA was extracted (Qiagen). RNA quality was assessed using the BioAnalyzer 2100™ (Agilent Technologies, Palo Alto, CA, USA) prior to reverse transcription-polymerase chain reaction (RT-PCR) analysis, and all samples with a 28S:18S ratio <1.2 were discarded. RT was performed with 0.5 μg of total RNA using the Cloned AMV Reverse Transcription Kit (Invitrogen, Paisley, UK), and cDNA quality was confirmed by PCR amplification of actin. Samples for which actin could not be amplified after 30 cycles of PCR were discarded. p53 isoform cDNA was amplified by two consecutive PCR assays (nested PCR) of 30 cycles each, and the PCR primers used were specific for each of the p53 isoforms analysed. The different primers used and their corresponding sequences are indicated in Table S1 in Additional file 1. To determine p53γ mutation status, the entire open reading frame of the isoform was sequenced using the Sanger method (BigDye Terminator, ABI 3730 Genetic Analyser; (Applied Biosystems, Warrington, UK) with the primers JWF (5'-AGCCAAGTCTGTGACTTGCA) and MP9ER (5'-TCTCCCAGGACAGCACAAACACG).

### *p53 *mutation analysis

The *p53 *mutation status was determined using 100 ng of genomic DNA extracted from homogenised frozen tissues as described previously using the AmpliChip p53 Test (Roche Diagnostics, Pleasanton, CA, USA) [21].

### Tumour grade, oestrogen receptor, progesterone receptor and HER2 status

Immunohistochemical staining was carried out on 4-μm sections of formalin-fixed, paraffin-embedded tumours with the mouse monoclonal anti-oestrogen receptor α (ER) antibody 6F11 (Novocastra Laboratories Ltd, Newcastle upon Tyne, UK), progesterone receptor (PR) antibody clone 16 (Novocastra Laboratories Ltd) and mouse monoclonal anti-HER2 antibody CB11 (Novocastra Laboratories Ltd). Additional analyses were performed according to histological tumour grade (graded by a specialist consultant breast pathologist); pathological tumour size (pT1, tumours <2 cm; pT2 and pT3 cancers, tumours ≥2 cm) [22]. ER status (ER negative 0 to 3 versus ER positive 4 to 18) was determined using the quickscore method [23]. Briefly, immunoreactivity scored semiquantitatively for both the intensity and the proportion of cells staining. Intensity was given scores from 0 to 3 (no staining = 0, light staining = 1, moderate staining = 2 and strong staining = 3) and proportion was given scores from 1 to 6 (0% to 4% = 1, 5% to 20% = 2, 21% to 40% = 3, 41% to 60% = 4, 61% to 80% = 5 and 81% to 100% = 6). The two scores were then multiplied to obtain the final result of 0 to 18. Human epidermal growth factor receptor 2 (HER2) scoring was performed as previously described [24].

### Statistical analysis

The primary outcomes in this study were breast cancer-specific overall survival (abbreviated to overall survival) and breast cancer-specific disease-free survival (abbreviated to disease-free survival or cancer recurrence throughout the text), and accordingly, non-breast cancer deaths were censored at the time of death (that is, at the time of death, the women were considered to have survived breast cancer but died as a result of other causes). Statistical analysis was performed using Minitab version 15.1.0.0 statistical software (Minitab Inc., PA 16801-3008, USA) for χ^2^, two-sided Fisher's exact test and Kaplan-Meier analyses. These univariate analyses test for associations between variables in a pairwise manner (for example, A versus B), but they do so without adjusting for influences exerted by other associated variables (for example, both A and B may be associated with confounding variables C, D and E, casting doubt on the validity of the relationship between A and B).

To clarify the univariate analyses and adjust for possible confounding variables, the selected variables were interrogated using the multivariate methods of binary logistic regression (BLR) with associated odds ratios (OR) and the Cox proportional hazards regression model (CR) with associated risk ratios (RR), both utilising the backwards stepwise elimination method. (For more detailed methods, read the "method" section in Additional file 2.)

In the tables of results for these multivariate analyses, the β value is a regression coefficient that indicates the strength of association between the predictor and response variables, where a large β indicates a strong association. A positive β indicates a positive association between the predictor and response variables, whilst a negative β indicates a negative association.

The OR is used to assess the risk of a particular outcome if a certain factor (or exposure) is present, indicating how much more likely it is that someone who is exposed to the factor under study will develop the outcome as compared to someone who is not exposed. If the odds are greater than 1, then the event is more likely to happen than not, whilst if the odds are less than 1, then the event is less likely. One 'reads' the risk ratios in precisely the same way.

The results of the univariate and multivariate analyses were consistent, and for clarity and brevity only the results of BLR, CR and Kaplan-Meier analyses are presented. Throughout the analyses the null hypothesis was rejected at an α level of 10% (*P *< 0.10), and observations considered to be marginal (that is, worthy of further analysis) for an α level between 5% and 10% (0.05 ≤ *P *≤ 0.10) and significant at 5% (*P *< 0.05). The *P *value represents the probability of error that is involved in accepting our observed result as valid. For example, *P *= 0.05 indicates that there is a 5% probability that the relation between the variables found in the sample occurred by chance.

## Results

### p53β and p53γ isoform expression in primary breast cancers

Cancers from 127 women (median age, 60.0 years; age range, 32.1 to 89.1 years) were examined. The majority of cancers were ductal carcinomas at 84% (107 of 127). Of these cases, 77% (98 of 127) were ER-positive, 62% (79 of 127) were PR-positive, 14% (17 of 119) were HER2-positive and 22% (28 of 127) had a tumour containing mutant *p53*. Approximately 50% (63 of 127) of the patients had axillary lymph node metastasis; tumours were grade 1 (16 cancers), grade 2 (48 cancers) or grade 3 (61 cancers), respectively. This patient population was therefore representative of symptomatic primary breast cancers in a Western country.

**Figure 1:**
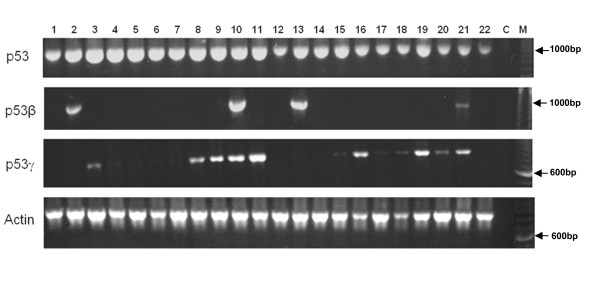
**p53β and p53γ mRNA are differentially expressed in primary breast tumours from patient to patient**. Total RNA from 127 primary breast tumours were provided by the Tayside Tissue Bank. RNA quality was assessed, and reverse transcription was performed as described in Materials and methods. The p53 cDNA were amplified by polymerase chain reaction (PCR) assays using primers specific for p53, p53β and p53γ, as shown in Table S1 in Additional file 1. Amplification of actin cDNA by PCR assay was used as a positive control. Tumour sample numbers are indicated. C, negative control; M, molecular marker.

Expression of p53β and p53γ was successfully analysed in the 127 primary breast cancers by using RT-PCR (Figure 1). On testing in triplicate, breast cancers consistently demonstrated p53β expression (36%; 46 of 127) and p53γ expression (37%; 47 of 127). Only 19% (24 of 127) of tumours expressed both p53β and p53γ.

Univariate statistical analysis determined that both p53β and p53γ were associated with clinical markers (data not shown). To clarify these associations and adjust for possible confounding variables, BLR analyses were performed to examine the associations of the various clinical markers with p53β and p53γ (Tables S2 and S3 in Additional file 1). p53β isoform expression was independently associated with p53γ expression (*P *= 0.008, BLR) (Table 1), and p53γ expression was independently associated with *p53 *mutation (*P *= 0.002, BLR) (Table 1). However, after adjusting for other associated clinical markers, p53β expression was not associated with *p53 *mutation (*P *= 0.970, BLR) (Table 1). For tumours bearing the *p53 *mutation, p53γ cDNA was directly sequenced using Sanger methods and was found to contain the same *p53 *mutation identified by the AmpliChip p53 Test, indicating that p53γ was expressed by tumour tissue from the same allele as the *p53 *mutation and not by stromal tissues. Most *p53 *mutations were hemizygous missense mutations affecting the DNA-binding domain of p53. Since the *p53 *gene was mutated at different codons in our cohort of breast cancer, there were not enough cases with the same *p53 *mutation for the statistical analysis.

**Table 1:** p53β and p53γ expression is associated with clinical markers

	All data			
Response variable	Predictor variable	β	*P *value	OR (95% CI)
p53β	p53γ	1.01	0.008	2.75 (1.29 to 5.84)
p53γ	p53m	1.47	0.002	4.33 (1.74 to 10.78)
	p53β	1.01	0.012	2.74 (1.24 to 6.04)
p53m	p53γ	1.74	0.002	5.70 (1.92 to 16.93)
	p53β	-0.02	0.970	0.98 (0.28 to 3.43)
	Tumour grade 3	2.85	<0.001	17.31 (4.02 to 74.45)
	ER	-2.74	0.019	0.06 (0.01 to 0.64)
	PR	2.51	0.032	12.27 (1.25 to 120.74)
				
Overall survival	p53m	-1.44	0.017	0.24 (0.07 to 0.78)
	PR	1.48	0.014	4.41 (1.34 to 14.45)
	Tumour size	-1.22	0.036	0.29 (0.09 to 0.93)
	p53γ	-0.18	0.783	0.83 (0.22 to 3.10)
	p53β	1.15	0.082	3.17 (0.87 to 11.60)
				
Cancer recurrence	p53m	1.25	0.013	0.29 (0.11 to 0.76)
	PR	-1.26	0.009	3.52 (1.37 to 9.04)
	p53γ	-0.28	0.636	1.33 (0.41 to 4.30)
	p53β	-0.74	0.198	2.10 (0.68 to 6.47)

Variables were analysed using binary logistic regression analysis utilising the backwards stepwise elimination method. Lymph node status, tumour grade, *p53 *mutation status (p53m), p53β, p53γ, human epidermal growth factor receptor 2 (HER2 or ErbB2), oestrogen receptor (ER) and progesterone receptor (PR) expression were included in the analyses as predictor variables. All independent significant associations between the predictor and response variables were identified (results of run 1). Dependent associations (results of runs 2, 3, 4, and so on) are omitted. Only results related to p53 and clinical outcome are presented. Results related to ER/PR, tumour grade and lymph node status are presented in Table S4 in Additional file 1. The β coefficients and the odds ratios (OR) with 95% confidence intervals (95% CI) are indicated.

### p53β and p53γ isoform expression, clinical and pathological associations

p53β expression was independently associated with ER status (*P *= 0.033, BLR) (Table S4 in Additional file 1), but not with PR status. p53γ expression was not associated with either ER or PR status. p53β and p53γ isoform expression was not associated with tumour type, menopausal status, age of cancer onset or HER2 status (data not shown).

As expected, *p53 *mutation was independently associated with cancer recurrence and death (*P *= 0.013 and *P *= 0.017, respectively; BLR) (Table 1). However, neither p53β nor p53γ isoform expression was associated with cancer recurrence (*P *= 0.198 and *P *= 0.636, respectively) (Table 1) or death (*P *= 0.082 and *P *= 0.783, respectively) (Table 1).

To determine whether the associations between *p53 *mutation and the various clinical markers were different in p53β- or p53γ-positive tumours, data were stratified by p53β and p53γ expression status. BLR analyses were performed to examine the associations between markers in the p53β- and p53γ-positive and p53β- and p53γ-negative cohorts (Table 2, and Tables S2 and S3 in Additional file 1).

**Table 2:** p53γ expression abolishes the association of p53 mutation status with poor prognosis

**p53γ**^**- **^**cohort**				
Response variable	Predictor variables	β	*P*	OR (95% CI)
Overall survival	p53m	-2.70	0.002	0.07 (0.01 to 0.37)
Cancer recurrence	p53m	2.77	0.001	0.06 (0.01 to 0.34)
				
p53γ^+ ^cohort				
Overall survival	Nothing associated			
Cancer recurrence	Nothing associated			

Variables were analysed using binary logistic regression analysis utilising the backwards stepwise elimination method. Lymph node status, tumour grade, *p53 *mutation status (p53m), p53β, p53γ, human epidermal growth factor receptor 2 (HER2 or ErbB2), oestrogen receptor (ER) and progesterone receptor (PR) expression were included in the analyses as predictor variables. All independent significant associations between the predictor and response variables were identified (results of run 1). Dependent associations (results of runs 2, 3, 4, and so on) are omitted. Only results related to p53 and clinical outcomes are presented. Results related to ER/PR, tumour grade and lymph node status are presented in Table S4 in Additional file 1. The β coefficient and the odds ratio (OR) with 95% confidence intervals (95% CI) are indicated.

Regarding p53β, *p53 *mutation status was marginally associated with cancer recurrence and death in the p53β-negative cohort (*P *= 0.059 and *P *= 0.072, respectively; BLR) (Table S2 in Additional file 1), while in the p53β-positive cohort *p53 *mutation status was not associated with death but was associated with cancer recurrence (*P *= 0.018, BLR; Table S2 in Additional file 1).

Regarding p53γ, *p53 *mutation status was independently associated with cancer recurrence and death in the p53γ-negative cohort (*P *= 0.001 and *P *= 0.002, respectively; BLR) (Table 2, and Table S3 in Additional file 1). Interestingly, *p53 *mutation status was not associated with cancer recurrence or death in the p53γ-positive cohort (*P *= 0.579 and *P *= 0.282, respectively; BLR) (Table 2, and Table S3 in Additional file 1), despite the greater proportion of grade 3 cancers with *p53 *mutations (61.5%; 16 of 26) in the p53γ-positive cohort compared with the p53γ-negative cohort (25.7%; 9 of 35). These data suggest that p53γ expression delineates two subpopulations of mutant *p53 *breast cancer patients with markedly different outcomes.

### p53β, p53γ and clinical outcome

To investigate the association between p53β or p53γ isoform expression and the clinical markers in relation to survival and cancer recurrence, we performed CR analyses that included p53β, p53γ, *p53 *mutation status and clinical markers (Table 3). These analyses demonstrated the expected associations between prognosis (death and cancer recurrence) and ER, PR, tumour grade, *p53 *mutation, HER2 or lymph node status (Table 3, rows a-f), but did not show any independent association for p53β or p53γ isoforms (Table 3, rows g and h).

**Table 3:** Cox regression analyses: p53 mutation status in p53 mutant breast cancer patients expressing p53γ is not associated with death and cancer recurrence

	Death					Recurrence				
ID	Run	Predictor	β	*P*	RR (95% CI)	Run	Predictor	β	*P*	RR (95% CI)
a	1	Tumour grade 3	2.12	0.005	8.33 (1.90 to 36.47)	1	PR	-0.99	0.021	0.37 (0.16 to 0.86)
b	2	PR	-1.36	0.011	0.26 (0.09 to 0.74)	1	p53m	1.06	0.012	2.87 (1.27 to 6.51)
c	2	p53m	1.1	0.026	3.00 (1.14 to 7.85)	2	Tumour grade 3	1.51	0.003	4.52 (1.60 to 12.11)
d	3	ER	-1.54	0.002	0.21 (0.08 to 0.55)	3	HER2	1.16	0.012	3.18 (1.29 to 7.83)
e	4	HER2	1.21	0.026	3.35 (1.16 to 9.65)	4	ER	-1.05	0.012	0.35 (0.16 to 0.79)
f	5^b^	Lymph nodes	1.19	0.037	3.30 (1.08 to 10.13)	5^b^	Lymph nodes	0.93	0.039	2.53 (1.05 to 6.09)
g	6^b^	p53β	-0.38	0.491	0.69 (0.24 to 2.00)	6^b^	p53β	-0.17	0.699	0.84 (0.35 to 2.03)
h	6^b^	p53γ	0.48	0.337	1.61 (0.61 to 4.29)	6^b^	p53γ	0.19	0.652	1.21 (0.52 to 2.81)
										
i	5^b^	p53m&p53β^+^	0.07	0.451	1.47 (0.54 to 3.99)	5^b^	p53m&p53β^+^	0.97	0.053	2.65 (0.99 to 7.10)
j	5^b^	p53m&p53γ^+^	0.31	0.626	1.36 (0.39 to 4.75)	5^b^	p53m&p53γ^+^	0.49	0.333	1.63 (0.61 to 4.36)
										
k	2	p53m&p53β^+ ^p53γ^-^	2.87	0.017	17.56 (1.66 to 186.15)	4	p53m&p53β^+^&p53γ^-^	1.71	0.021	5.51 (1.29 to 23.57)
l	5^b^	p53m&p53γ^+^&p53β^-^	0.78	0.298	2.19 (0.50 to 9.59)	5^b^	p53m&p53γ^+^&p53β^-^	0.31	0.679	1.36 (0.32 to 5.78)

^a^Variables were analysed using Cox proportional hazards regression model utilising the backwards stepwise elimination method. Lymph node status, tumour grade, *p53 *mutation status (p53m), p53β, p53γ, human epidermal growth factor receptor 2 (HER2 or ErbB2), oestrogen receptor (ER) and progesterone receptor (PR) expression were included in analyses as predictor variables. The run number refers to the run in which the predictor variable was deemed to be statistically significantly associated with the response variable (death or recurrence) and thereafter was excluded from further runs. Rows a-f refer to the statistically significant results in the first iteration of analyses without interaction variables. Of the variables that were not associated with the response variables, p53β and p53γ are included in the table (rows g and h), but others are omitted for clarity and brevity. Rows i-l refer to results of Cox regression analyses for the interaction predictor variables shown in the presence of all the above predictor variables (data not shown for clarity and brevity). The β coefficient and the risk ratio (RR) with 95% confidence intervals (95% CI) are indicated; ^b^Final run in a given set of analyses.

Further, to determine the degree of interdependence between the variables p53β, p53γ and *p53 *mutation with respect to survival and cancer recurrence, we aggregated these variables into combined variables and reran the CR analyses. We thus formed a binary variable (p53m&p53β) that was positive when p53β was expressed and the *p53 *gene was mutated, but negative otherwise. Similarly, we formed a binary variable (p53m&p53γ) that was positive when p53γ was expressed and the *p53 *gene was mutated, but negative otherwise. The results of using such combined variables (Table 3, row i and row j) allowed us to determine that *p53 *mutation is no longer associated with death or cancer recurrence when p53β or p53γ is expressed (Table 3, compare row c with row i or row j). This effect was independent of ER status and therefore independent of endocrine therapy (in this study, all ER-positive patients were treated with tamoxifen 20 mg for 5 years as standard adjuvant therapy). Moreover, there was also no significant difference in the ER status of patients bearing *p53 *mutations between the p53γ-positive and p53γ-negative cohorts (*P *= 0.254, Fisher's exact test).

Furthermore, given the association between p53β and p53γ expression (Table 1), we performed CR analysis to determine the combined effects of p53γ and *p53 *mutation in the absence of p53β and reciprocally the combined effects of p53β and *p53 *mutation in the absence of p53γ. We formed a binary variable (p53m&p53β^+ ^p53γ^-^) that was positive when p53β was expressed in the absence of p53γ expression and the *p53 *gene was mutated (Table 3, row k). We also formed a binary variable (p53m&p53γ^+ ^p53β^-^) that was positive when p53γ was expressed in the absence of p53β expression and the *p53 *gene was mutated (Table 3, row l). These analyses revealed that *p53 *mutation in patients expressing p53β but not p53γ retained the association with death and cancer recurrence (Table 3, compare row c, row i and row k), while *p53 *mutation in patients without p53β but with p53γ was not associated with death and cancer recurrence (Table 3, row c, row j and row l).

Therefore, the apparent abrogation of the association of *p53 *mutation with poor prognosis in the p53γ-positive population (but not in the p53β-positive population) indicates that only p53γ allows the identification of a subpopulation of breast cancer patients expressing mutant *p53 *with a better prognosis than expected.

### p53β, p53γ expression, p53 mutation and clinical outcomes

Using Kaplan-Meier log-rank analyses, patients with mutant *p53 *breast cancer had significantly worse disease-free survival and overall survival than those with wild-type p53 breast cancer (Kaplan-Meier log-rank test, χ^2 ^= 10.51, 1 *df*, *P *= 0.001; and χ^2 ^= 6.55, 1 *df*, *P *= 0.010, respectively) (Figure S1 in Additional file 3), with a more than three times increased risk of recurrence and death (hazard ratio (HR) = 3.48 and HR = 3.16, respectively). Expression of p53β or p53γ was not associated with cancer recurrence (Kaplan-Meier log-rank test, χ^2 ^= 0.05, 1 *df*, *P *= 0.817; Figure S2 in Additional file 4, bottom; and χ^2 ^= 0.15, 1 *df*, *P *= 0.694; Figure S3 in Additional file 5, bottom, respectively) or with overall survival (Kaplan-Meier log-rank test, χ^2 ^= 0.37, 1 *df*, *P *= 0.544; Figure S2 in Additional file 4, top; and χ^2 ^= 0.31, 1 *df*, *P *= 0.575; Figure S3 in Additional file 5, top, respectively).

**Figure 2:**
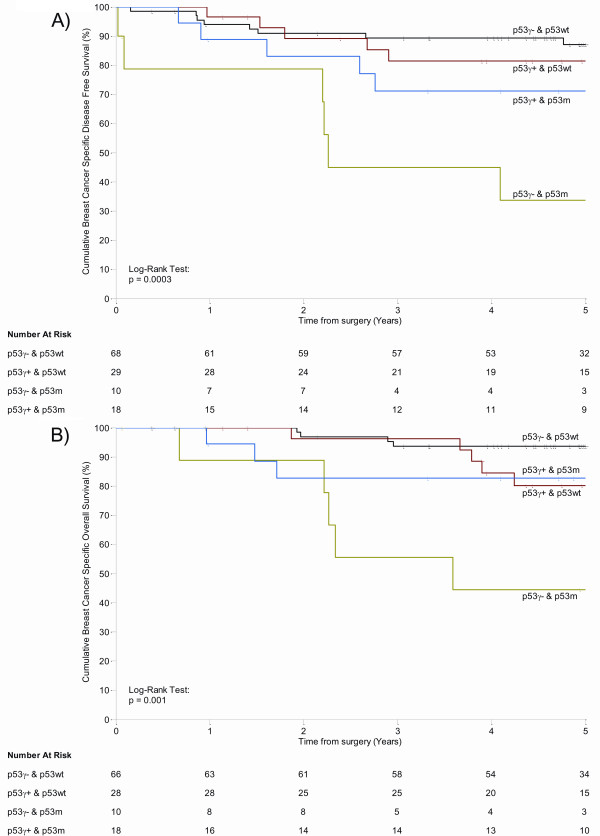
**p53 mutant breast cancer patients expressing p53γ have disease-free survival and overall survival comparable to that of patients bearing wild-type (wt) p53 breast cancer**. Nonparametric Kaplan-Meier plots for p53γ expression and *p53 *gene mutation of **(A) **disease-free survival (*n *= 125) and **(B) **overall survival (*n *= 122). Censored cases are indicated by 'l' on the curves. *P *values are indicated.

Patients bearing mutant *p53 *tumours and expressing the p53γ isoform had disease-free survival and overall survival that were not different from patients bearing wild-type p53 tumours, with a low comparative risk of recurrence and a similar risk of death (HR = 1.72 and HR = 1.04, respectively) (Figure 2A: Kaplan-Meier log-rank test, χ^2 ^= 0.76, 1 *df*, *P *= 0.384; and Figure 2B: Kaplan-Meier log-rank test, χ^2 ^< 0.01, 1 *df*, *P *= 0.958, respectively). However, patients bearing mutant *p53 *tumours without p53γ isoform expression had a high risk of recurrence and subsequent high risk of death (HR = 7.21 and HR = 11.23, respectively) compared with patients bearing wild-type p53 tumours (Figure 2; Kaplan-Meier log-rank test: χ^2 ^= 18.33, 1 *df*, *P *< 0.001; and χ^2 ^= 20.70, 1 *df*, *P *< 0.001, respectively). Consistent with the CR analysis, the Kaplan-Meier log-rank analyses indicate that p53γ allows the identification of a subpopulation of breast cancer patients expressing mutant *p53 *with a better prognosis than expected.

## Discussion

The p53 network is thought to be ubiquitously altered in human cancers, either through mutation of the *p53 *gene or through inactivation of p53 protein [1]. In breast cancer, it has been difficult to link p53 mutation status to therapeutic response and clinical outcome, suggesting that additional factors may affect the p53 network. We previously reported that the *p53 *gene expresses at least nine p53 protein isoforms in normal human tissue, including p53β and p53γ, which are differentially expressed in breast cancer as in other types of cancer [13-19]. In this study, we report the analysis of expression of p53β and p53γ in relation to clinical and pathological markers and disease outcome in a cohort of 127 randomly selected primary breast tumours.

In our cohort, p53β expression was detected in 36% of the primary breast tumours and was associated with ER expression but not with disease outcome. p53γ expression was detected in 37% of primary breast tumours and was associated with *p53 *gene mutation. The potentially clinically significant finding was that p53γ expression allowed discrimination between two subpopulations of patients bearing mutant *p53 *tumours: (1) patients bearing mutant *p53 *cancer and expressing p53γ who had disease-free survival and overall survival as good as patients with wild-type p53 and (2) patients bearing mutant *p53 *tumours without detectable p53γ isoform expression who had a particularly poor prognosis. Importantly, there was no significant difference in the ER status of patients bearing *p53 *mutations between the p53γ-positive and p53γ-negative cohorts (*P *= 0.254, Fisher's exact test). Therefore, the better outcomes of the breast cancer patients expressing p53γ and mutant *p53 *are not due to endocrine therapy in ER-positive cancers.

We have chosen to perform this analysis without previously classifying tumours according to immunohistochemical phenotype (luminal (A and B), HER2, basal (triple-negative: ER^-^, PR^-^, HER^-^) and unclassified) because, in our cohort, the low number of tumours in each immunohistochemical phenotype did not allow us to perform CR and Kaplan-Meier log-rank analyses to investigate p53 isoform expression in relation to clinical outcome. Indeed, among the 85 luminal tumours (ER^+^, PR^+ ^and HER^-^), only 10 tumours expressed mutant *p53*. This low number of *p53 *mutations did not allow us to find a significant statistical association between p53 isoform expression and *p53 *mutation. By contrast, regarding patients who were not in the luminal group (basal and unclassified tumours), 13 of 16 tumours with a *p53 *mutation expressed p53γ (81%), whilst 14 of 18 tumours with wild-type *p53 *did not express p53γ (78%). This finding confirms that p53γ expression is associated with p53 mutation status. Regarding patients who were in the basal group (triple-negative), there was a significant positive association between p53γ expression and *p53 *mutation, with 6 of 7 tumours with a p53 mutation expressing p53γ (86%), whilst 9 of 10 tumours with wild-type *p53 *did not express p53γ (90%). The result in nonluminal patients is consistent with the results obtained without classifying tumours according to immunohistochemical phenotype. Of note, the lack of association in luminal patients between p53γ expression and *p53 *mutation is probably due to the low number of *p53 *mutations in this breast cancer subtype.

By sequencing p53γ cDNA in breast tumours expressing mutant *p53*, we noted that p53γ cDNA contained the same mutation as the *p53 *gene, indicating that p53γ was expressed by the tumour cells and not by cells from the stroma. Therefore, this finding suggests either that the mutant p53γ isoform has an intrinsic activity abrogating the poor prognosis associated with *p53 *mutation or that p53γ is just an inactive marker of better outcomes for mutant *p53 *breast cancer patients. Future investigations will seek to determine the biological and biochemical activities of mutant p53γ and its interplay with mutant p53 in tumour cells.

We did not differentiate between the different categories of p53 mutations (nonsense mutations, missense mutations, 'DNA-contact' mutations or 'conformational' mutations), as there were not enough cases in each p53 mutation category for confident statistical analysis. However, in larger breast cancer cohorts, it would be interesting to take the different p53 mutation categories and molecular subtypes of breast cancer into account to refine the statistical analysis.

Currently, p53γ expression can be specifically detected only by PCR. From a clinical utility perspective, it would be useful to analyse p53β and p53γ expression by using immunohistochemistry. The mouse monoclonal antibodies DO-1 and DO-7 recognise p53, p53β and p53γ, but not the other p53 isoforms. The rabbit or sheep polyclonal p53 antibodies (CM1 and Sapu, respectively) raised against recombinant full-length human p53 protein recognize all p53 isoforms, while the KJC8 antibody recognises specifically all p53β isoforms (that is, p53β, Δ40p53β and Δ133p53β). However, we have been unable to stain paraffin-embedded sections using the KJC8 antibody. Since p53β and p53γ can be localised in both the nucleus and the cytoplasm, we have attempted to determine by performing immunohistochemistry on paraffin-embedded breast tumour sections using DO-1 or CM1 p53 antibodies whether p53β or p53γ expression is associated with cytoplasmic or nuclear staining. There was no significant association between p53 cytoplasmic or nuclear staining by DO-1 or CM1 and p53β or p53γ expression. Pending the generation of isoform-specific antibodies, p53 immunostaining on tumour sections should be interpreted with caution and should be complemented by PCR analysis to determine p53 isoform mRNA expression in tumours.

Treatment influences were not identified in this analysis, although no taxane, cisplatin or trastuzumab therapy was administered to the patients studied, and anthracycline-based chemotherapy was the standard agent used during the sample accrual period. *p53 *mutation may be associated with resistance to several chemotherapy agents; but *p53 *mutant breast cancer may be more sensitive to taxanes, at least in the neoadjuvant setting [5-10], and the predictive value of *p53 *mutational status in breast cancer remains controversial [3,4]. The influence of the p53γ isoform in the setting of clinical trials such as the neoadjuvant European Organisation for Research and Treatment of Cancer (EORTC) 10994 Trial, which is testing the association between *p53 *mutation and taxane versus anthracycline therapy, merits consideration and would provide potential validation of the association of the p53γ isoform with *p53 *mutation and prognosis in the setting of a randomized, controlled trial. In addition, since mutant *p53 *cancers are generally of basal or triple-negative phenotype, the influence of the p53 isoforms on platinum therapies and poly(ADP-ribose) polymerase inhibitors in appropriate clinical trials would be of interest. Meanwhile, the apparently dominant effects of the p53γ isoform influencing the p53 network may provide an explanation for the conflicting literature regarding the clinical associations between mutant *p53 *and breast cancer and issue a warning that clinical decisions made on the basis of *p53 *mutation status alone may need to be approached with caution.

## Conclusions

In this report, the expression of the p53β and p53γ isoforms was examined in relation to clinical and pathological markers, p53 mutation and disease outcome in a cohort of 127 randomly selected primary breast tumours. We determined that p53β and p53γ isoform expression is associated, respectively, with ER status and p53 mutation. p53β or p53γ isoform expression is not independently associated with overall survival or disease-free survival. On the basis of multivariate analyses and Kaplan-Meier analyses, we determined that the breast cancer patient group expressing both mutant *p53 *and the p53γ isoform has a disease-free and overall survival as good as the patient group bearing wild-type p53 breast cancer. Conversely, patients expressing only mutant *p53 *without p53γ isoform expression had a particularly poor prognosis. The p53γ isoform may explain the inconsistent relationship between *p53 *mutation and breast cancer reported in the literature.

## Abbreviations

BLR: binary logistic regression; CI: confidence interval; CR: Cox proportional hazards regression model; ER: oestrogen receptor; OR: odds ratio; PR: progesterone receptor; RT-PCR: reverse transcription-polymerase chain reaction; RR: relative risk.

## Competing interests

The authors declare that they have no competing interests.

## Authors' contributions

JCB, DPL and AT conceived and designed the study. CP and LJ conducted the pathological review. AD, MK, KF, MA and ML performed the experiments. MK, LB, PQ, ACP, JCB and AT contributed to data analysis and interpretation. LB, MK, KF, JCB and AT contributed to the writing of the report.

## Supplementary Material

Additional file 1 **Supplementary Tables**. **Table S1. Primers used for amplification of p53 isoforms and actin by RT-PCR (nested PCRs)**. List of the primers used to amplify each human p53 mRNA isoform specifically. **Table S2. Binary logistic regression analyses of the p53β-positive and p53β-negative cohorts**. Multivariate analysis of p53β expression in relation to clinical markers and clinical outcomes. **Table S3. Binary logistic regression analyses of the p53γ-positive and p53γ-negative cohorts**. Multivariate analysis of p53γ expression in relation to clinical markers and clinical outcomes. **Table S4. p53β is associated with oestrogen receptor status**. Binary logistic regression analyses including lymph node status, tumour grade, p53 mutation status (p53m), p53β, p53γ, HER2 (erbB2), oestrogen receptor (ER) and progesterone receptor (PR) expression as predictor variables. Click here for file

Additional file 2 **Detailed statistical analysis**. Explanation of the statistical analysis. Click here for file

Additional file 3 **Figure S1**. Analysis of *p53 *mutation status in relation to breast cancer-specific overall survival and disease-free survival of primary breast cancer patients. Nonparametric Kaplan-Meier plots of **(A) **disease-free survival (that is, 100% minus percentage of cancer recurrence) (*n *= 125) and **(B) **overall survival (*n *= 122) in relation to *p53 *gene mutation status. Censored cases are shown as 'l' on the curves. *P *values are based on log-rank tests. Click here for file

Additional file 4 **Figure S2**. Analysis of p53β expression in relation to breast cancer-specific overall survival and disease-free survival of primary breast cancer patients. Nonparametric Kaplan-Meier plots of **(A) **disease-free survival (that is, 100%-percentage of cancer recurrence) (*n *= 125) and **(B) **overall survival (*n *= 122) in relation to p53β expression. Censored cases are shown as 'l' on the curves. *P *values are based on log-rank tests. Click here for file

Additional file 5 **Figure S3**. Analysis of p53γ expression in relation to breast cancer-specific overall survival and disease-free survival of primary breast cancer patients. Nonparametric Kaplan-Meier plots of **(A) **disease free survival (that is, 100%- percentage of cancer recurrence) (*n *= 125) and **(B) **overall survival (*n *= 122) in relation to p53γ expression. Censored cases are shown as 'l' on the curves. *P *values are based on log-rank tests. Click here for file
