# Supplementary material for: p53 mutant breast cancer patients expressing p53γ have as good a prognosis as wild-type p53 breast cancer patients
Source: Breast Cancer Res. 2011 Jan 20;13(1):R7. doi: 10.1186/bcr2811 (PMC3109573; doi:10.1186/bcr2811)
Supplement: Additional file 1 — Supplementary Tables. Table S1. Primers used for amplification of p53 isoforms and actin by RT-PCR (nested PCRs). List of the primers used to amplify each human p53 mRNA isoform specifically. Table S2. Binary logistic regression analyses of the p53β-positive and p53β-negative cohorts. Multivariate analysis of p53β expression in relation to clinical markers and clinical outcomes. Table S3. Binary logistic regression analyses of the p53γ-positive and p53γ-negative cohorts. Multivariate analysis of p53γ expression in relation to clinical markers and clinical outcomes. Table S4. p53β is associated with oestrogen receptor status. Binary logistic regression analyses including lymph node status, tumour grade, p53 mutation status (p53m), p53β, p53γ, HER2 (erbB2), oestrogen receptor (ER) and progesterone receptor (PR) expression as predictor variables. [file bcr2811-S1.DOC]

**Table S1:** Primers for amplification of p53 isoforms and Actin by RT-PCR (nested PCRs).

| **mRNA** | **PCR** | **Primer name and targeted region** | **5’ – 3’ sequence** |
| --- | --- | --- | --- |
| **p53** | **I** | **e2.1 (F)** (exon2) | GTCACTGCCATGGAGGAGCCGCA |
|  |  | **RT1 (R)** (exon11) | GACGCACACCTATTGCAAGCAAGGGTTC |
|  | **II** | **e2 (F)** (exon2) | ATGGAGGAGCCGCAGTCAGAT |
|  |  | **RT2 (R)** (exon11) | ATGTCAGTCTGAGTCAGGCCCTTCTGTC |
| **p53β** | **I** | **e2.1 (F)** (exon2) | GTCACTGCCATGGAGGAGCCGCA |
|  |  | **RT1 (R)** (exon11) | GACGCACACCTATTGCAAGCAAGGGTTC |
|  | **II** | **e2 (F)** (exon2) | ATGGAGGAGCCGCAGTCAGAT |
|  |  | **p53β (R)** (exon9b) | TTTGAAAGCTGGTCTGGTCCTGA |
| **p53γ** | **I** | **e2.1 (F)** (exon2) | GTCACTGCCATGGAGGAGCCGCA |
|  |  | **RT1 (R)** (exon11) | GACGCACACCTATTGCAAGCAAGGGTTC |
|  | **II** | **e2 (F)** (exon2) | ATGGAGGAGCCGCAGTCAGAT |
|  |  | **p53γ (R)** (exon9b) | TCGTAAGTCAAGTAGCATCTGAAGG |
| **Actin** | **actin5 (F)** | | ATCTGGCACCACACCTTCTACAATGAGCTGCG |
|  | **actin3 (R)** | | CGTCATACTCCTGCTTGCTGATCCACATCTGC |

For each p53 isoform, the nested PCR assay was performed as two consecutive PCR reactions (I, II) with two separate primer pairs as indicated. (F): Forward, (R): Reverse.

**Table S2:** Binary Logistic Regression Analyses of the p53β positive and negative cohorts


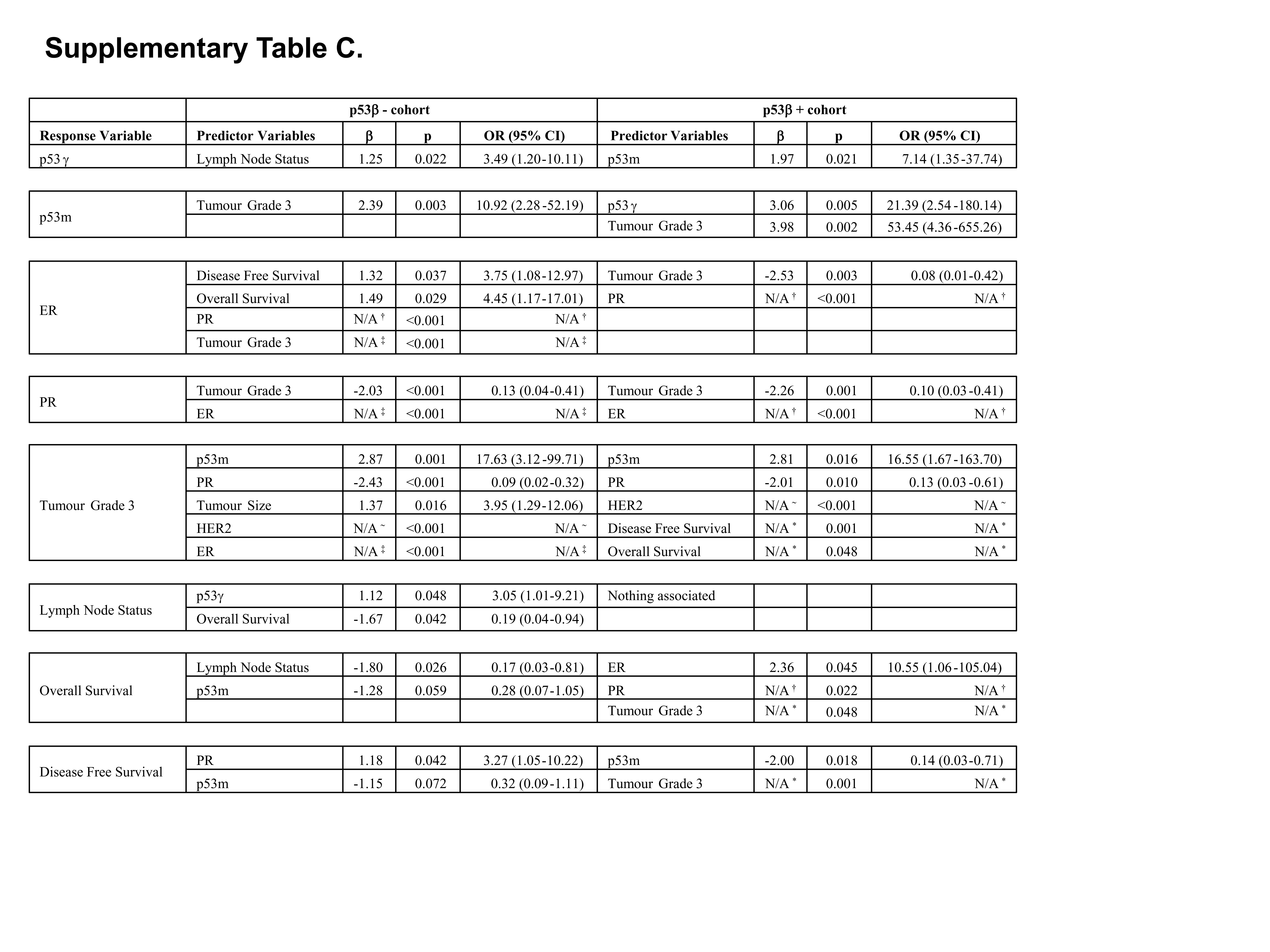


Variables were analyzed by Binary Logistic Regression utilising the backwards step-wise elimination method. Lymph node status, tumour grade, p53 mutation status (p53m), p53β, p53γ, HER2 (erbB2), Estrogen Receptor (ER) and Progesterone Receptor (PR) expressions were included in analyses as predictor variables. All independent significant associations between the predictor and response variables were identified (results of run 1). Dependent associations (results of runs 2, 3, 4, etc.) have been omitted. The β coefficient and the Odds Ratio (OR) with 95% confidence intervals (CI) are indicated. Notes: †all PR positive patients were ER positive and all survived; all patients with low grade tumours were ‡ER positive in the p53β negative cohort and *remained disease free and survived in the p53β positive cohort; ~all HER2 positive patients had grade 3 tumours.

**Table S3:** Binary Logistic Regression Analyses of the p53γ positive and negative cohorts


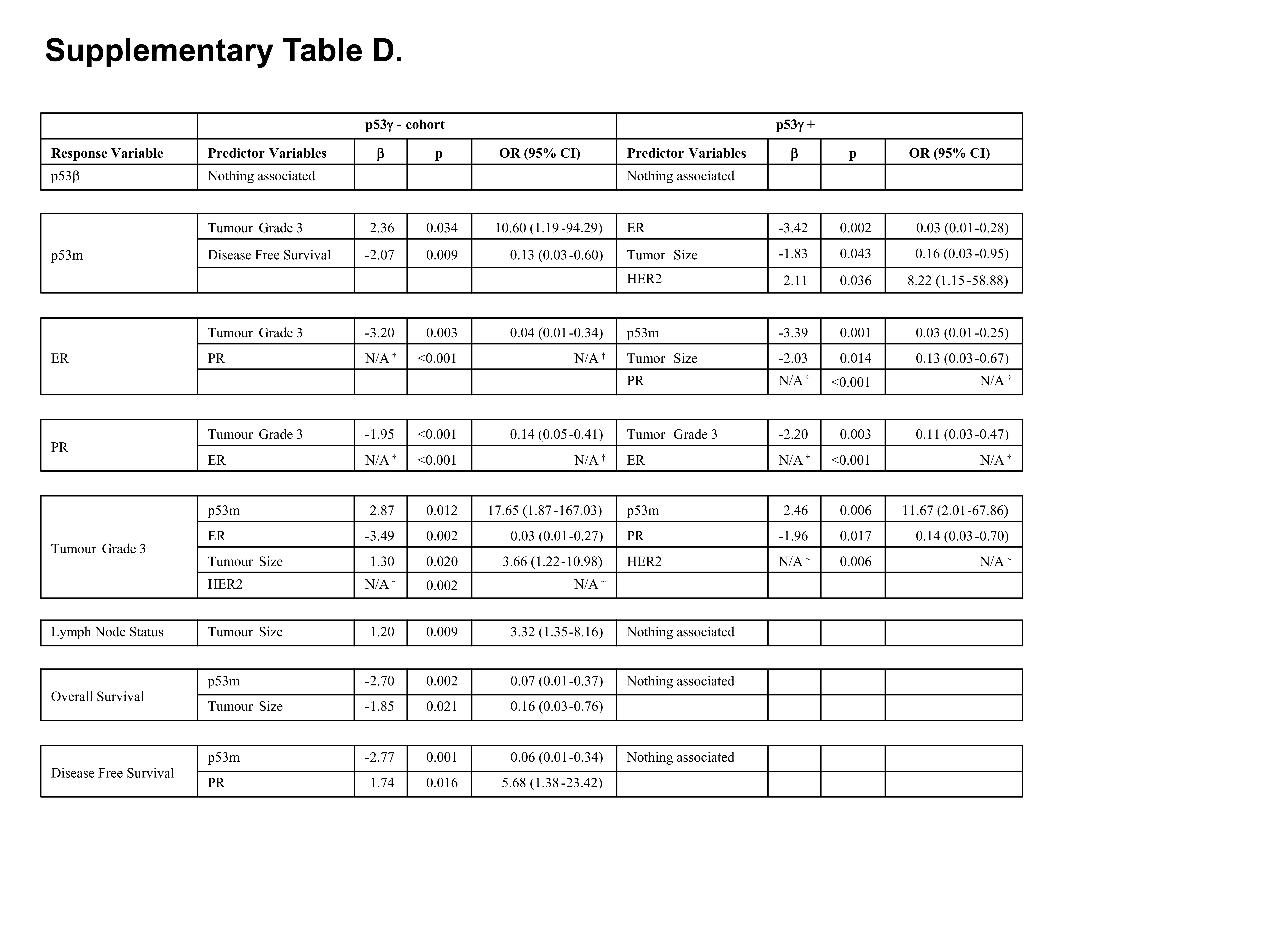


Variables were analyzed by Binary Logistic Regression utilising the backwards step-wise elimination method. Lymph node status, tumour grade, p53 mutation status (p53m), p53β, p53γ, HER2 (erbB2), Estrogen Receptor (ER) and Progesterone Receptor (PR) expressions were included in analyses as predictor variables. All independent significant associations between the predictor and response variables were identified (results of run 1). Dependent associations (results of runs 2, 3, 4, etc.) have been omitted. The β coefficient and the Odds Ratio (OR) with 95% confidence intervals (CI) are indicated. Notes: †There were no patients that were ER negative and PR positive; ~all HER2 positive patients had grade 3 tumours.

**Table S4:** p53β is associated to Estrogen Receptor status.

|  | | | | |
| --- | --- | --- | --- | --- |
|  | **All Data** | | | |
| **Response Variable** | **Predictor Variables** | **β** | **p** | **OR (95% CI)** |
| ER | p53β | -1.10 | 0.033 | 0.33 (0.12-0.91) |
|  | Tumour Grade 3 | -3.19 | <0.001 | 0.04 (0.01-0.19) |
|  | PR | N/A^‡^ | <0.001 | N/A^‡^ |
|  |  |  |  |  |
| PR | Tumour Grade 3 | -2.05 | <0.001 | 0.13 (0.06-0.30) |
|  | ER | N/A^‡^ | <0.001 | N/A^‡^ |
|  |  |  |  |  |
| Tumour Grade 3 | p53m | 2.79 | <0.001 | 16.22 (4.22-62.31) |
|  | PR | -2.17 | <0.001 | 0.11 (0.05-0.29) |
|  | HER2 | N/A^†^ | <0.001 | N/A^†^ |

Variables were analysed by Binary Logistic Regression utilising the backwards step-wise elimination method. Lymph node status, tumour grade, p53 mutation status (p53m), p53β, p53γ, HER2 (erbB2), Estrogen Receptor (ER) and Progesterone Receptor (PR) expressions were included in analyses as predictor variables. All independent significant associations between the predictor and response variables were identified (results of run 1). Dependent associations (results of runs 2, 3, 4, etc.) have been omitted. Only results related to p53 and clinical outcome are presented. The β coefficient and the Odds Ratio (OR) with 95% confidence intervals (CI) are indicated.
