# Supplementary material for: p53 mutant breast cancer patients expressing p53γ have as good a prognosis as wild-type p53 breast cancer patients
Source: Breast Cancer Res. 2011 Jan 20;13(1):R7. doi: 10.1186/bcr2811 (PMC3109573; doi:10.1186/bcr2811)
Supplement: Additional file 2 — Detailed statistical analysis. Explanation of the statistical analysis. [file bcr2811-S2.DOC]

**Additional Methods**

**Statistical analysis**

Multivariate analyses allow multiple predictor variables to be tested simultaneously for associations with a ‘target’ (response) variable whilst adjusting for the comparative effects of all other predictor variables. Such analyses clarify which variables are (1) independently associated with the target variable (they exert a direct influence), (2) dependently associated (they exert an indirect influence via other variables) or (3) not associated at all (no influence is exerted). All multivariate analyses presented here utilised a backwards step-wise elimination method. Briefly, all predictor variables were initially present in ‘run 1.1’ of the model. At its conclusion, the most non-significant (highest non-significant p-value) variable was eliminated, and the model re-ran (‘run 1.2’). This continued until ‘run 1.x’, when all remaining predictor variables were significantly and independently associated with the response variable (or all predictor variables were eliminated). The predictor variables that were eliminated were not independently – but they may be dependently – associated with the response variable (i.e. they exert an indirect influence). Therefore, at ‘run 2.1’, we omitted all the independently associated predictor variables and re-ran to ‘run 2.y’. This continued for runs 3, 4, etc., until no more runs were possible. At the conclusion of this process, predictor variables that remained at the end of run 1 were independently associated with the response variable, those present at the end of runs 2, 3, 4, etc., were dependently associated, whilst all variables that were eliminated from the final run were not associated with the predictor variable.
