# Supplementary figures and images for: p53 mutant breast cancer patients expressing p53γ have as good a prognosis as wild-type p53 breast cancer patients
Source: Breast Cancer Res. 2011 Jan 20;13(1):R7. doi: 10.1186/bcr2811 (PMC3109573; doi:10.1186/bcr2811)

# Supplementary Figure 1.

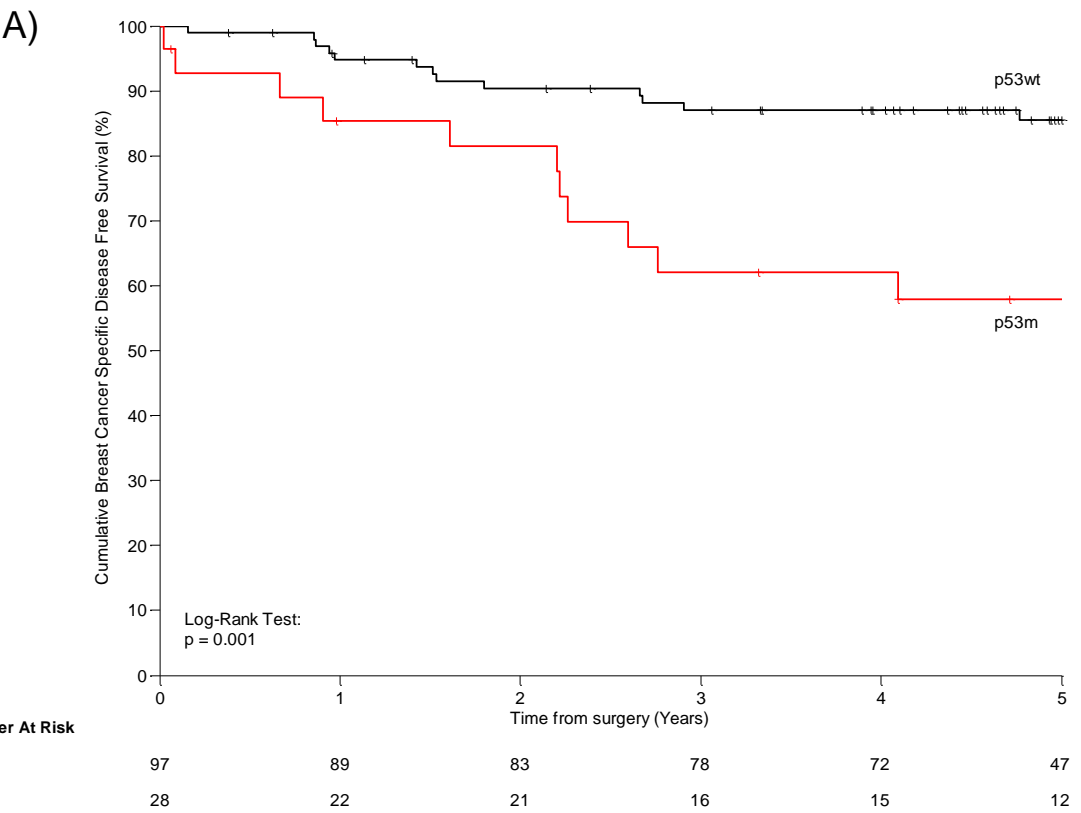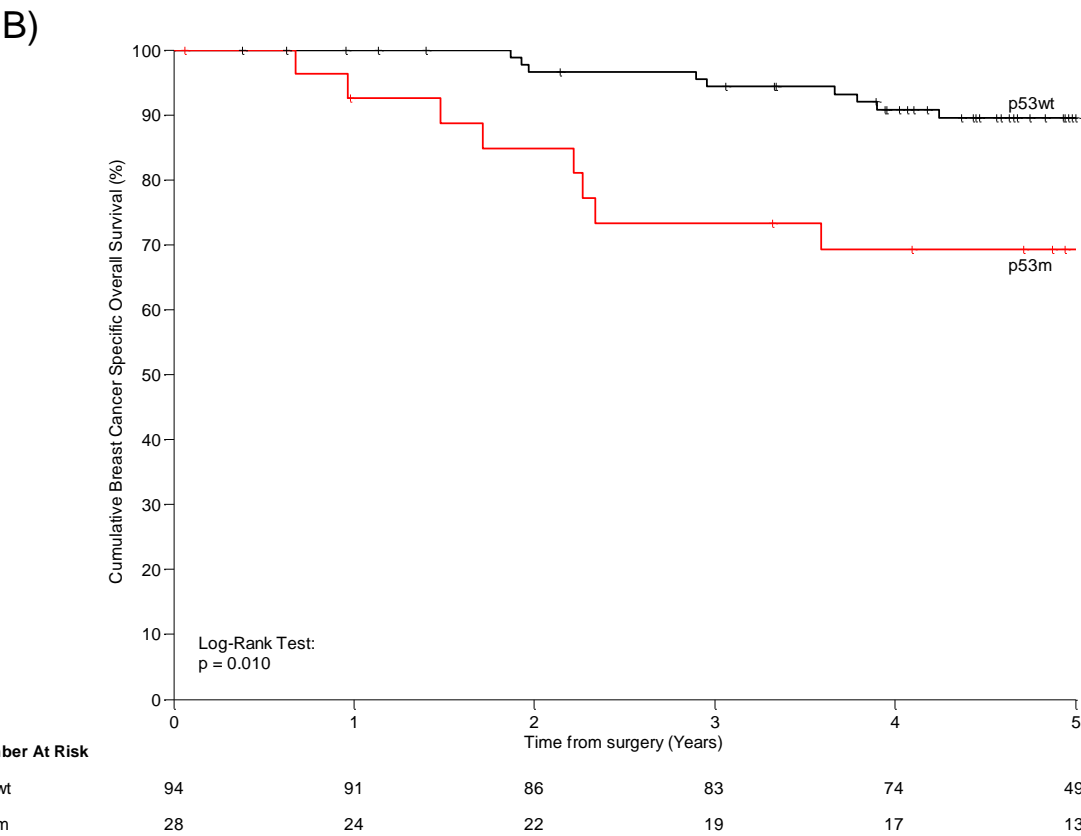

Supplement: Additional file 3 — Figure S1. Analysis of p53 mutation status in relation to breast cancer-specific overall survival and disease-free survival of primary breast cancer patients. Nonparametric Kaplan-Meier plots of (A) disease-free survival (that is, 100% minus percentage of cancer recurrence) (n = 125) and (B) overall survival (n = 122) in relation to p53 gene mutation status. Censored cases are shown as 'l' on the curves. P values are based on log-rank tests. [file bcr2811-S3.PDF]

Supplementary Figure 2.

A)

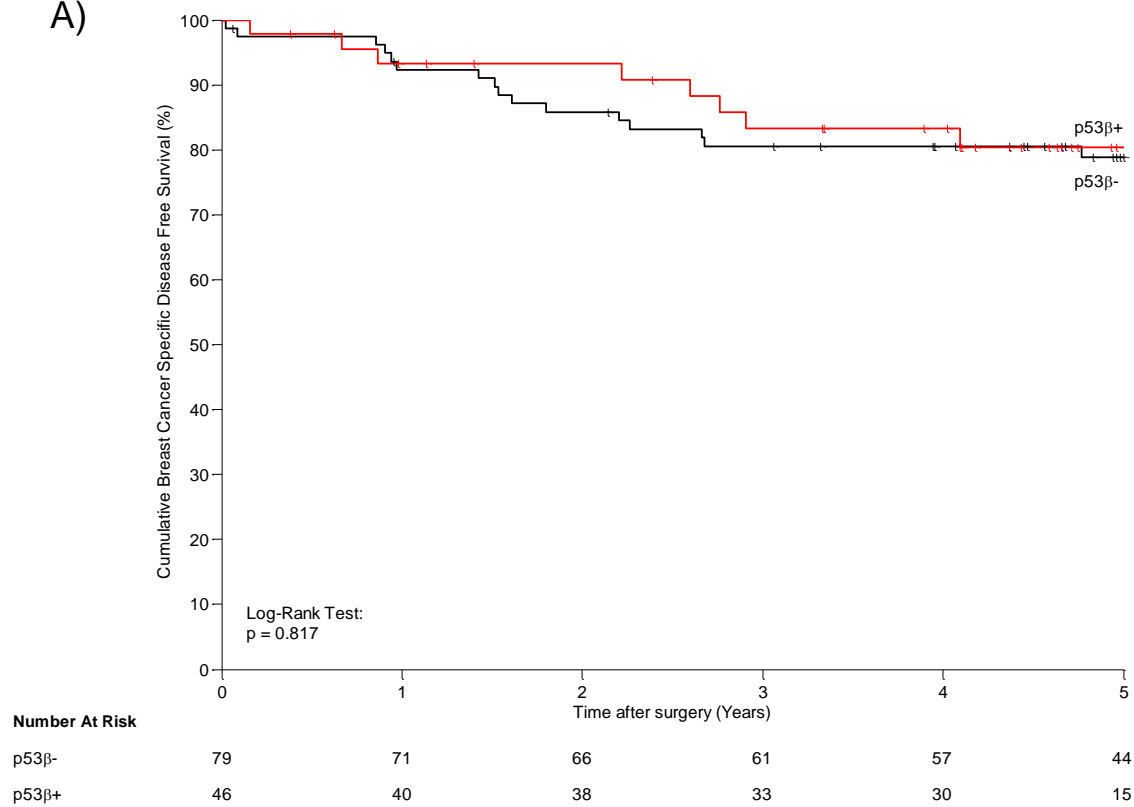

B)

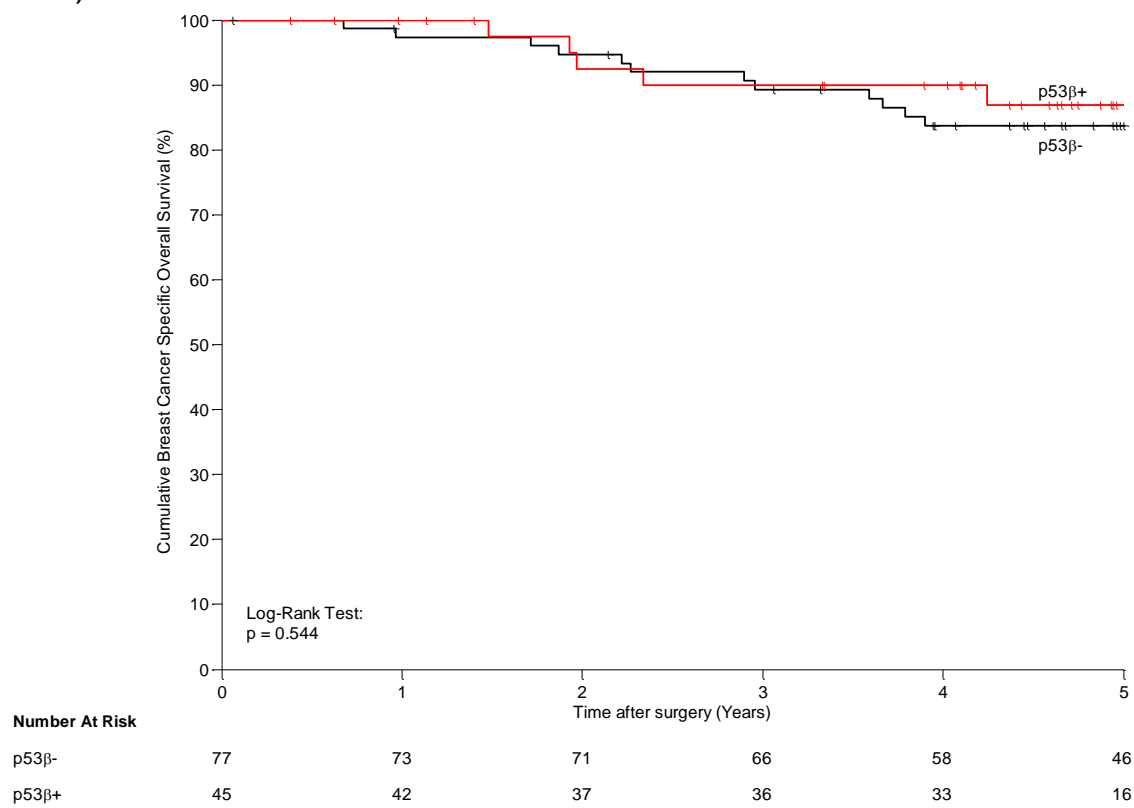

Supplement: Additional file 4 — Figure S2. Analysis of p53β expression in relation to breast cancer-specific overall survival and disease-free survival of primary breast cancer patients. Nonparametric Kaplan-Meier plots of (A) disease-free survival (that is, 100%-percentage of cancer recurrence) (n = 125) and (B) overall survival (n = 122) in relation to p53β expression. Censored cases are shown as 'l' on the curves. P values are based on log-rank tests. [file bcr2811-S4.PDF]

# Supplementary Figure 3.

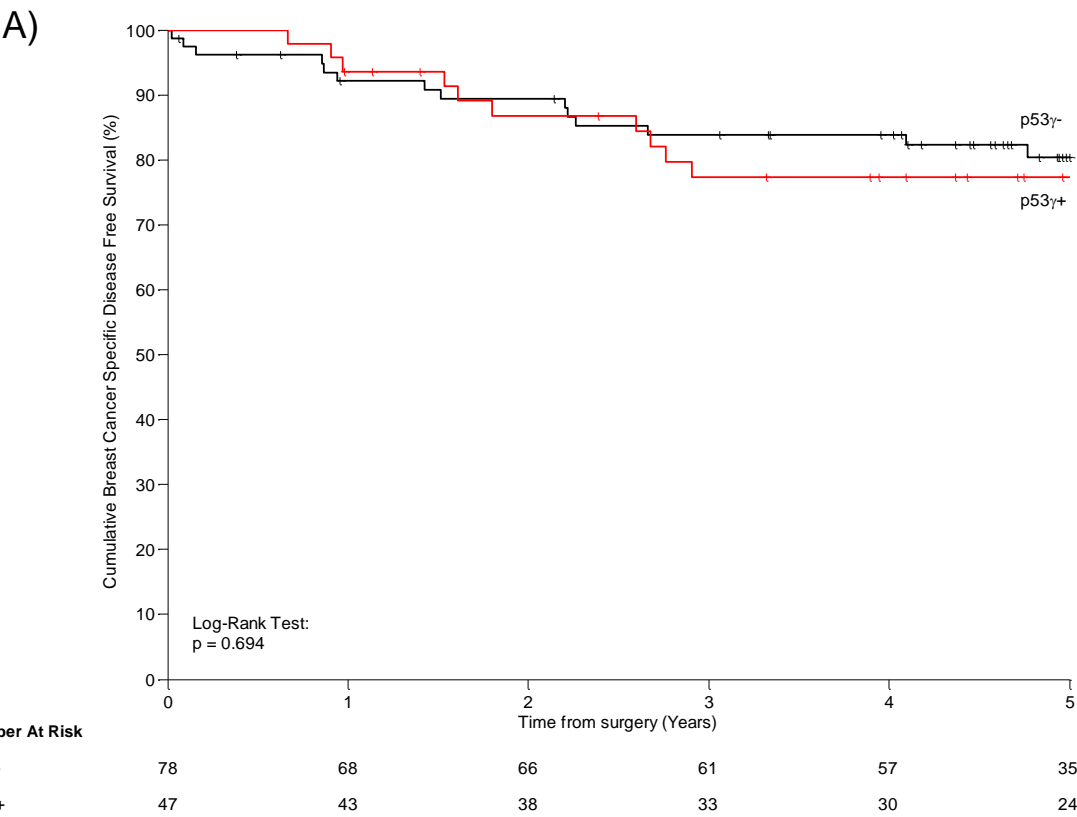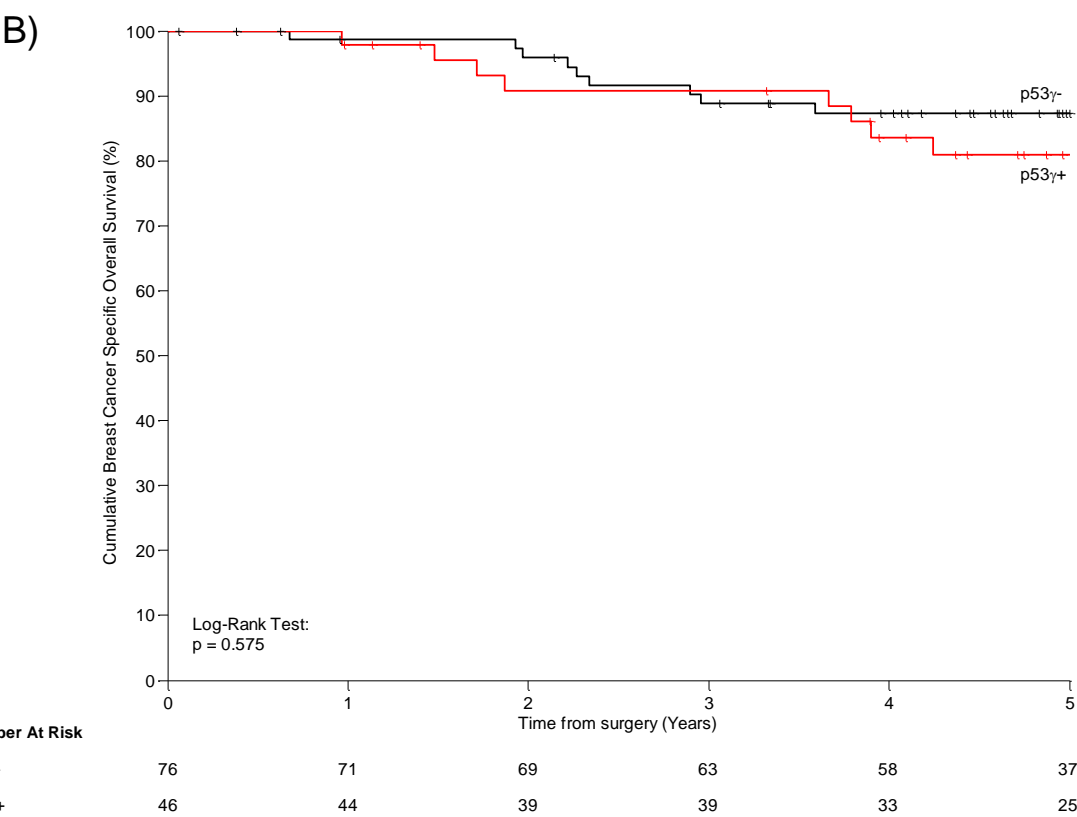

Supplement: Additional file 5 — Figure S3. Analysis of p53γ expression in relation to breast cancer-specific overall survival and disease-free survival of primary breast cancer patients. Nonparametric Kaplan-Meier plots of (A) disease free survival (that is, 100%- percentage of cancer recurrence) (n = 125) and (B) overall survival (n = 122) in relation to p53γ expression. Censored cases are shown as 'l' on the curves. P values are based on log-rank tests. [file bcr2811-S5.PDF]
